# MXene (Ti_3_C_2_T*_x_*) Functionalized Short Carbon Fibers as a Cross-Scale Mechanical Reinforcement for Epoxy Composites

**DOI:** 10.3390/polym13111825

**Published:** 2021-05-31

**Authors:** Lu Liu, Guobing Ying, Cheng Sun, Huihua Min, Jianxin Zhang, Yinlong Zhao, Dong Wen, Ziying Ji, Xing Liu, Chen Zhang, Cheng Wang

**Affiliations:** 1 Department of Materials Science and Engineering, College of Mechanics and Materials, Hohai University, Nanjing 211100, China; liulu201709@163.com (L.L.); suncheng2019@126.com (C.S.); zhangjianxin2019@126.com (J.Z.); loong1016@126.com (Y.Z.); wendonghhu@163.com (D.W.); jiziying0411@163.com (Z.J.); liuxing200003@163.com (X.L.); zhangchh2015@126.com (C.Z.); wangch@hhu.edu.cn (C.W.); 2 Electron Microscope Lab, Nanjing Forestry University, Nanjing 210037, China; hhmin@njfu.edu.cn

**Keywords:** MXene Ti_3_C_2_T_x_, functionalization, short carbon fibers, epoxy-matrix composites, cross-scale reinforcement, mechanical properties

## Abstract

The surface modification technology of carbon fibers (CFs) have achieved considerable development, and it has achieved great success in improving the interfacial shear strength (IFSS) of the polymer matrix. Among them, MXene (Ti_3_C_2_T*_x_*) functionalized CFs have been proven to improve the interface performance significantly. Unfortunately, the results on the microscopic scale are rarely applied to the preparation of macroscopic composite materials. Herein, the process of MXene functionalized CFs were attempted to be extended to short carbon fibers (SCFs) and used to strengthen epoxy materials. The results show that the cross-scale reinforcement of MXene functionalized SCFs can be firmly bonded to the epoxy matrix, which significantly improves the mechanical properties. Compared to neat epoxy, the tensile strength (141.2 ± 2.3 MPa), flexural strength (199.3 ± 8.9 MPa) and critical stress intensity factor (*K*_IC_, 2.34 ± 0.04 MPa·m^1/2^) are increased by 100%, 67%, and 216%, respectively.

## 1. Introduction

Carbon fibers (CFs) have a series of advantages, such as high specific strength, high specific modulus, high fatigue resistance, high corrosion resistance, and low linear coefficient of thermal expansion, so it is an ideal structural material for reinforcing polymer matrix [1,2,3,4]. In order to improve the wettability of CFs surface with polymer to form a solid interface, researchers have used various methods to modify the surface of CFs to improve the surface polarity (polar component [5]) or surface roughness (dispersion component [6]) of carbon fibers [7,8,9,10,11,12,13,14]. Our previous work [15] reported a method of using MXene (T_3_C_2_T*_x_*) to functionalized CFs, which the MXene connected to acid-treated CFs (ACFs) through hydrogen bonding. In this way, the polar component and dispersion component of the CFs were increased at the same time, and the IFSS was increased by 186%.

MXene is a transition metal carbide and nitride with a graphene-like structure 2D materials [16,17,18]. Due to the unique 2D layered structure, large specific surface area, excellent mechanical properties, rich surface functional groups, and light transmittance [19,20,21], excellent electrical conductivity [22], MXene has been widely used in the field of reinforced polymer composites [23,24,25,26,27,28,29,30]. The general formula of MXene is M_n+1_X_n_T*_x_* (*n* = 1, 2, 3 or 4), M refers to early transition metals, and X refers to C or N element [31,32,33,34]. The T*_x_* in the formula represents the surface terminations, such as O, OH, F, and/or Cl, which are bonded to the outer M layers [35,36,37,38]. The surface activity of MXene is extremely high, and it can form a strong chemical bond with the epoxy matrix [39]. Therefore, MXene is an ideal material for modifying the interface properties of CF/epoxy composites.

Although the interface combination of modified CFs and polymer have been improved, it is still challenging to re-weave them into bundles or cloth for further applications. The CFs products pre-woven into the finished products are inaccessible to the modified substance due to the overlap between the fibers, which greatly reduces the modification effect. Therefore, short carbon fibers (SCFs) or surface-modified SCFs have attracted attention in the field of polymer reinforcement [40,41,42,43,44,45]. Reinforcing the epoxy resin with low-load SCFs have practical significance because the viscosity of the resin can be maintained at an acceptable level.

The present paper attempts to extend the successful surface modification method of continuous CFs to SCFs for epoxy reinforcement. ACFs were prepared into acid-treated SCFs (ASCFs) to prepare ASCFs/epoxy composites with different ASCFs contents. Simultaneously, ASCFs were functionalized by MXene (T_3_C_2_T*_x_*) to obtain the surface-modified cross-scale reinforcement. After finding out the most suitable load of ASCFs, the epoxy-matrix composites with the same amount of ASCFs functionalized by MXene were prepared. The results show that an appropriate amount of ASCFs can improve the mechanical properties of epoxy resin. On this basis, the ASCFs functionalized by MXene can further enhance the mechanical properties. The characterization results of the fracture indicate that the MXene functionalized ASCFs have a stronger bond with the epoxy matrix. Compared to the neat epoxy, the tensile strength, flexural strength and critical stress intensity factor (*K*_IC_) of composites can be increased to 100%, 67% and 216% at the maximum, which has a good application prospect.

## 2. Experimental

### 2.1. MXene Functionalization of SCFs

**Figure 1:**
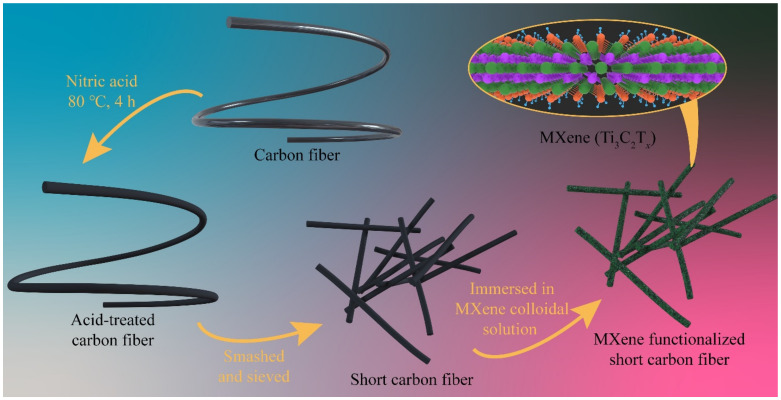
Schematic representation of MXene functionalized ASCFs.

Comprehensive details regarding all of the materials and the preparation of Ti_3_AlC_2_ and Ti_3_C_2_T_x_ are provided in our earlier papers [39,46,47]. At first, the carbon fibers (T300C, 3K, diameter 7 μm, density 1.76 g·cm^−1^, Toray Industries Inc, Tokyo, Japan) were cleaned with refluxed acetone (Sinopharm Chemical Reagent limited corporation, Shanghai, China) at 60 °C for 96 h to remove any polymer sizing and pollutants and yield untreated CFs. The untreated CFs were then oxidized in concentrated nitric acid (HNO_3_, 68 wt.%, Sinopharm Chemical Reagent Limited Corporation, Shanghai, China) at 80 °C for 4 h. Subsequently, the carbon fibers were taken out and washed several times with de-ionized water (resistivity > 18 MΩ cm, prepared in a laboratory) until pH approached 7. Next, the obtained fibers were dried in a vacuum furnace to yield ACFs. The ACFs were mashed and ground in an agate mortar, and passed through a 325-mesh sieve 3 times. The sieved powders were washed with de-ionized water, then centrifuged at 3500 rpm for 5 min. After the precipitate was thoroughly dried, the ASCF with a clean surface and an average length of ~20 μm were obtained. Finally, the ASCF was immersed in a solution with a concentration of MXene of 1 mg mL^−1^ for 15 min, washed with de-ionized water after being taken out and dried in a vacuum oven to obtain MXene functionalized ASCFs, as shown in Figure 1.

### 2.2. Synthesis of Composite

DGEBA (SINOPEC Baling Company, Yueyang, China) and MTHPA (Tianjin Chemical Co., Tianjin, China) were mixed in a mass ratio of 100:85. After the mixture was softened at 80 °C, ASCFs were added and ultrasonicated for 1 h. The 0.3 wt.% 2,4,6-Tris (dimethylaminomethyl) phenol (Tianjin Chemical Co., Tianjin, China) was added after the ultrasonic treatment, and then it was cured in a vacuum drying oven at 90 °C for 1 h and 110 °C for 4 h. According to the different addition amounts of ASCFs, epoxy-matrix composites with mass fractions of 1%, 2%, 3% and 4% were prepared and denoted as C1/epoxy, C2/epoxy, C3/epoxy and C4/epoxy, respectively. According to the results of tensile and flexural tests, the cross-scale reinforcements contain 2 wt.% ASCFs were added to DGEBA/MTHPA to prepare the MXene-functionalized ASCF/epoxy composite and denoted as C2M/epoxy.

### 2.3. Characterization

Thermogravimetric analyses (TGA, NETZSCH STA 449F3, Selb, Germany) of neat epoxy and composites were carried out under a nitrogen atmosphere from 25 to 790 °C at a heating rate of 10 °C min^−1^. The storage modulus and loss factor (tanδ) as a function of temperature were determined via single cantilever mode of the dynamic mechanical analyzer (DMA, TA Q800, New Castle, DE, USA) in the temperature range from 25 to 200 °C at 3 °C min^−1^ under air atmosphere, frequency of 1 Hz, and maximum amplitude of 0.1%. The specimen dimension was kept at 30.0 mm long × 10.0 mm wide × 5 mm thick. The glass transition temperature was obtained by using the maximum tanδ. The Micro-morphology of specimens was gold-coated and observed at 5 kV by scanning electron microscope (SEM, JEOL JSM-7600F, Tokyo, Japan). Moreover, energy dispersive spectrometer (EDS, Oxford INCA X-Act, Oxford, UK) was utilized to analyze the mapping of elements.

The tensile and flexural tests were performed at room temperature using an Instron 3367 mechanical testing machine (Instron Co. Ltd., Canton, MA, USA) following ISO 527-1:1993 and ISO-178-2010, respectively. Standard dumbbell-shaped specimens (75.0 mm long × 12.5 mm wide × 2.0 mm thick) with a length of the narrow region of 25 mm were prepared for tensile testing at a rate of 1.0 mm min^−1^. Rectangular specimens (60.0 mm long × 8.0 mm wide × 3.0 mm thick) were used for flexural testing and loaded with a span of 48 mm at a crosshead speed of 2.0 mm min^−1^. The elastic modulus was calculated by the ratio of the stress difference (σ_2_–σ_1_) to the corresponding strain difference (ε_2_–ε_1_), i.e., ε_2_ = 0.25% and ε_1_ = 0.05%. Similarly, the flexural modulus was also calculated by the ratio of the stress difference (σ_fM2_–σ_fM1_) to the corresponding strain difference (ε_fM2_–ε_fM1_), i.e., ε_fM2_ = 0.25% and ε_fM1_ = 0.05%. At least five specimens were tested under each set of conditions. The flexural strength (σ_fM_) and flexural strain (ε_fM_) was calculated by using Equation (1) [48] and Equation (2) [48] as follows:

(1) $$\sigma_{\mathrm{fM}}=\frac{3FL}{2bh^{2}}$$

(2) $$\varepsilon_{\mathrm{fM}}=\frac{6sh}{L^{2}}$$

where *F* is the maximum load of the load-displacement curve for bending specimens, *L* is the span, *b* is the width of the sample, *s* is the deflection, *h* is the thickness of the sample.

The fracture toughness values of the composites were determined following ISO 13586:2000 standard using single-edge-notched bend (SENB) specimens (60.0 mm long × 8.0 mm wide × 3.0 mm thick). A sharp notch was machined at the midpoint of each specimen (4 mm in depth), and a natural pre-crack was generated by tapping a new razor blade into the notch. SENB specimens were also tested by Instron 3367 using a three-point-bending rig. Owing to the brittle nature of epoxy, the test speed was set to 0.5 mm min^−1^ to achieve sufficient loading time before the end of each test. The critical stress intensity factor ($K_{IC}$) was calculated by using Equation (3) [49] as follows:

(3) $$K_{IC}=f\left(a/w\right)\frac{F_{Q}}{h\sqrt{w}}$$

where $F_{Q}$ is the maximum load of the load-displacement curve for SENB specimens, h is the thickness of the specimen, w is width, and a denotes a sharp crack of length between 0.45 w and 0.55 w. The $f\left(a/w\right)$ is related to the geometry of the sample and can be calculated by using Equations (4) and (5) [49] as follows:

(4) $$f\left(a/w\right)=6\alpha^{1/2}\frac{1.99-\alpha \left(1-\alpha \right)\left(2.15-3.93\alpha +2.7\alpha^{2}\right)}{\left(1+2\alpha \right){\left(1-\alpha \right)}^{3/2}}$$

(5) α=a/w

The critical energy release rate (*G*_IC_) was calculated as Equation (6) [50]:

(6) $$G_{IC}=K_{IC}^{2}\left(\frac{1-\nu^{2}}{E}\right)$$

where *E* is the elastic modulus of the MXene/epoxy composites, *ν* denotes Poisson’s ratio of DGEBA/MTHPA system, and the value of 0.29 was used [51].

## 3. Results and Discussion

**Figure 2:**
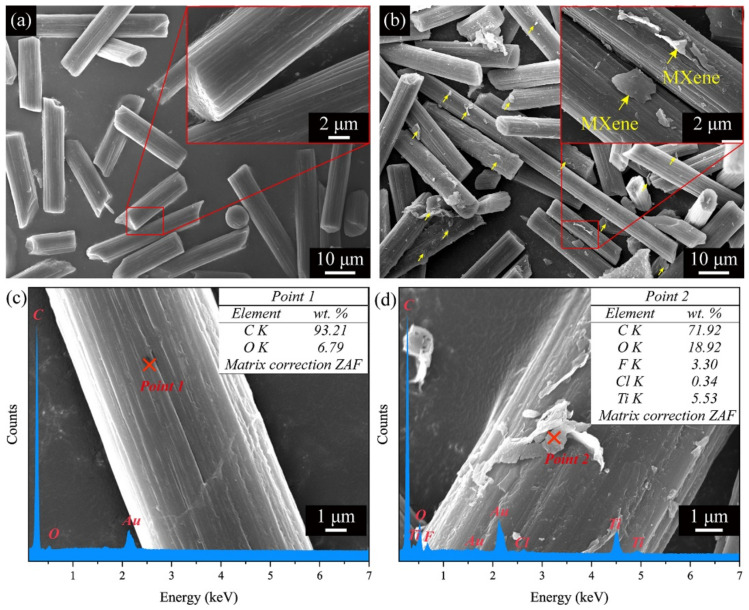
SEM images for (**a**) ASCFs and (**b**) MXene functionalized ACSFs. High-magnification SEM images and EDS results of (**c**) ASCFs and (**d**) MXene functionalized ACSFs.

The SEM images in Figure 2a,b show the morphology of ASCFs and MXene functionalized ACSFs, respectively. As shown in Figure 2a, the diameter and the length of the ASCFs prepared in this work are approximately 7 μm and 10–40 μm (average ~20 μm), respectively. The surfaces of the ASCFs are clean and have ravine-like characteristics after acid treatment [6]. In our previous work [15], the optimal solution concentration (1 mg mL^−1^) was determined when using MXene to functionalize ASCFs, so MXene does not accumulate too much on ASCFs at this concentration, as shown in Figure 2b. MXene is covered in a thin layer on most ASCFs to form cross-scale reinforcements, and MXene is warped in some places. As shown in Figure 2c,d, ASCFs and MXene functionalized ACSFs exhibited totally different surface morphologies, and EDS data confirmed the composition of the scaly wrinkles presented on the surface of ASCFs as Ti_3_C_2_T_x_ layers.

**Figure 3:**
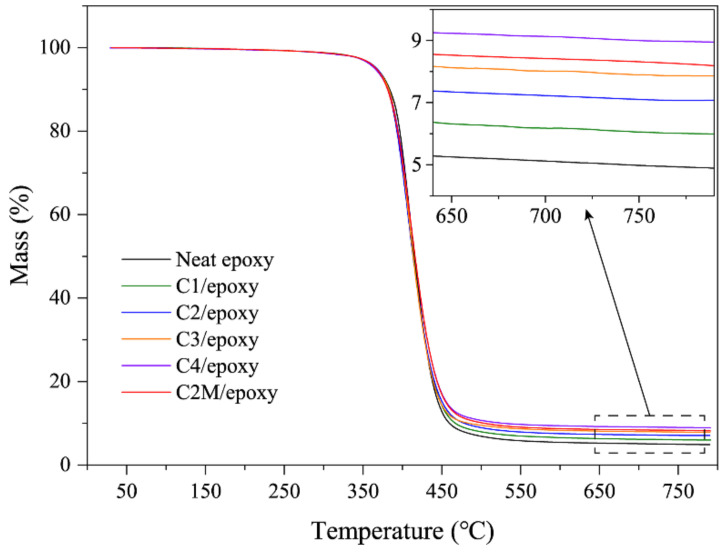
TGA plots of neat epoxy, ASCF/epoxy composites and MXene functionalized ASCF/epoxy composites under a nitrogen atmosphere.

TGA was utilized to investigate the thermal stability of each composite under a nitrogen atmosphere (Figure 3). All TGA curves exhibited one main degradation stage. The sharp mass loss in the range of 350–450 °C is mainly due to the decomposition of the epoxy matrix. The residual masses of all ASCF/epoxy composite samples at 790 °C are proportional to the ASCF’s load. The residual mass of C2M/epoxy is slightly larger than that of C2/epoxy. Besides the increase of residual mass due to the addition of MXene itself, the epoxy cannot be completely decomposed at a temperature of 800 °C, then MXene causes more char adhesion residue [52,53].

**Figure 4:**
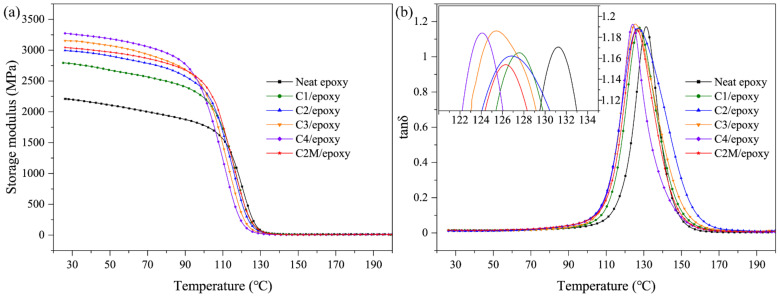
Dynamic mechanical properties of neat epoxy, ACSF/epoxy composites and MXene functionalized ACSF/epoxy composites: (**a**) storage modulus and (**b**) loss angle tangent.

Storage modulus is an index reflecting the elastic properties and influenced by the interfacial interactions between the filler and resin matrix of polymer composites [54]. As shown in Figure 4a, the incorporation of ASCFs leads to the increased storage modulus in the low-temperature range (25–70 °C). The addition of rigid fillers increases the storage modulus. In the range of room temperature to 140 °C, the storage modulus of C2M/epoxy is slightly higher than that of C2/epoxy at the same temperature. This phenomenon shows that the strength of the MXene functionalized ASCF/epoxy interface is stronger than that of the ASCF/epoxy interface, and the loads transfer from the epoxy-matrix to the ASCFs are more efficient.

Figure 4b illustrates the loss angle tangent (tanδ) of ASCF/epoxy and MXene functionalized ASCF/epoxy composites, and the temperature at maximum tanδ value reflects the glass transition temperature (T_g_). Compared to the neat epoxy, the T_g_ values of the composite decrease with the addition of reinforcement. Polymer composites have a large relaxation temperature range [55,56,57,58]. Reinforcements restrict the slippage of adjacent epoxy chains through strong interface bonding, making the connections around the reinforcement closer and reducing the cross-linking density of the epoxy network of the whole composite. Although the ASCFs content of C2M/epoxy and C2/epoxy are the same, the T_g_ of the former is slightly lower, which further proves the influence of solid interface on the cross-linked network. The storage modulus value at room temperature and T_g_ are shown in Table 1.

**Figure 5:**
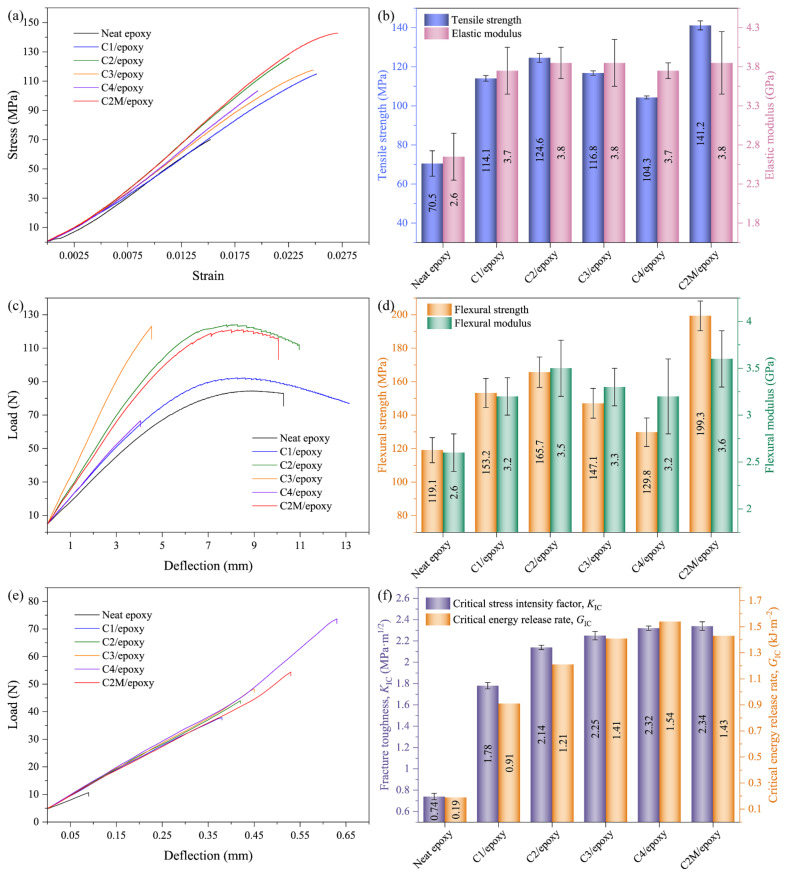
(**a**) Typical stress-strain curves in tension, (**b**) elastic modulus and tensile strength, (**c**) typical load-deflection curves in bending, (**d**) flexural modulus and flexural strength, (**e**) typical load-deflection curves in fracture toughness, (**f**) fracture toughness and critical energy release rate values of fracture toughness of ACSF/epoxy composites and MXene functionalized ACSF/epoxy composites.

Figure 5a,d exhibit the typical stress-strain curves in tension, tensile strength, elastic modulus, typical load-deflection curves in bending, flexural strength and flexural modulus of neat epoxy, ASCF/epoxy and MXene functionalized ASCF/epoxy composites at different filler loadings, respectively. It can be found that the addition of ASCFs enhances the mechanical properties of the composites when compared to neat epoxy. Increasing the ASCFs’ content first shows an increasing trend of properties, followed by a decrease. At the ASCFs’ content of 2 wt.%, all mechanical properties of the ASCF/epoxy composites reach maximum values. On this basis, the mechanical properties of C2M/epoxy composites are further improved. Compared to the C2/epoxy, tensile strength (124.6 MPa) is increased by 13% (141.2 MPa), while the flexural strength (165.7 MPa) is increased by 20% (199.3 MPa), respectively. Compared to neat epoxy, the mechanical properties are significantly improved, tensile strength and elastic modulus are increased by 100% and 46%, while the flexural strength and flexural modulus are increased by 67% and 38%, respectively. The mechanical properties for neat epoxy and its composites with different filler loadings are summarized in Table 2. Considering the enhancement effects of commercially available SCFs [43,44,59,60], MXene [39,61], the enhancements from the addition of MXene functionalized ACSFs are also comparable, as shown in Table 3.

**Figure 6:**
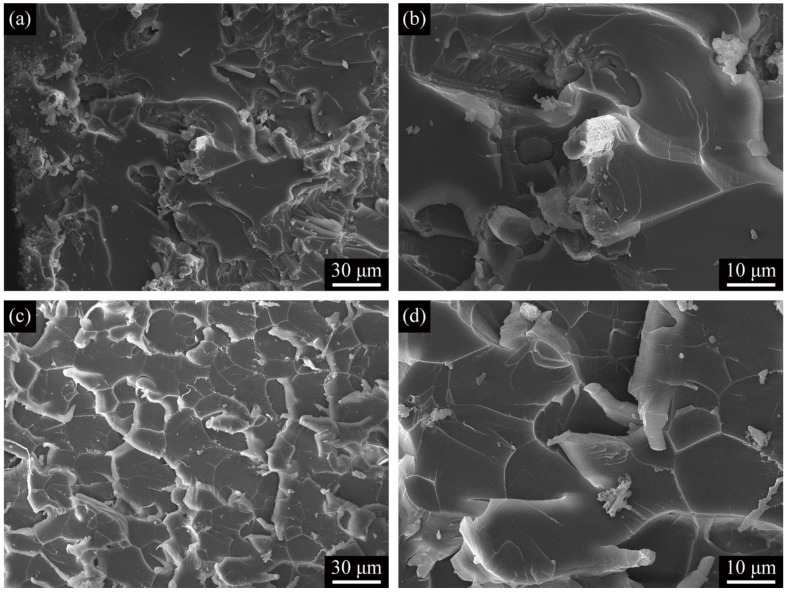
(**a**) Low-magnification and (**b**) high-magnification SEM images of tensile fracture surfaces of C2/epoxy; (**c**) Low-magnification and (**d**) high-magnification SEM images of tensile fracture surfaces of C2M/epoxy.

SEM was used to compare the fracture behavior of ASCF/epoxy composites and MXene functionalized ASCF/epoxy composites after the tensile test (Figure 6). The tensile fracture surfaces of C2/epoxy and C2M/epoxy composites both exhibit multi-planar features with many tortuous cracks, which indicates that the reinforcement phase caused the deflection of crack propagation. The crack deflection process can arouse off-plane loading and generate new fracture surfaces, increasing the required strain energy for crack propagation. It should be noted that there are many debonding ASCFs in the fracture section of C2/epoxy (Figure 6a,b); however, there is no debonding reinforcement on the fracture section of C2M/epoxy (Figure 6c,d). This failure mode observation indirectly reflects the solid interface interaction between the cross-scale reinforcement and the epoxy matrix, which was due to the ASCFs functionalized by MXene. For one thing, the wrinkled MXene layers change the surface roughness of the carbon fiber and enhance the mechanical interlock [54]; for another thing, the rich polar functional groups on the surface of MXene not only have strong hydrogen bonding with epoxy resin [15], but also can form a chemical bond with epoxy resin matrix [39].

Fracture toughness of ASCF/epoxy composites with different filler loadings and C2M/epoxy composites were evaluated by the SENB method, as shown in Figure 5f. The fracture toughness value is positively correlated with the amount of ASCFs added. C2M/epoxy has the largest fracture toughness value (2.34 MPa·m^1/2^), which is 9% higher than C2/epoxy (2.14 MPa·m^1/2^), and is significantly 216% higher than neat epoxy (0.74 MPa·m^1/2^). The propagation of crack is severely hindered through the fillers, which leads to the dissipation of higher fracture energy in composites. Further, the critical energy release rate (*G*_IC_) of C2M/epoxy has increased dramatically by ~650% than neat epoxy.

**Figure 7:**
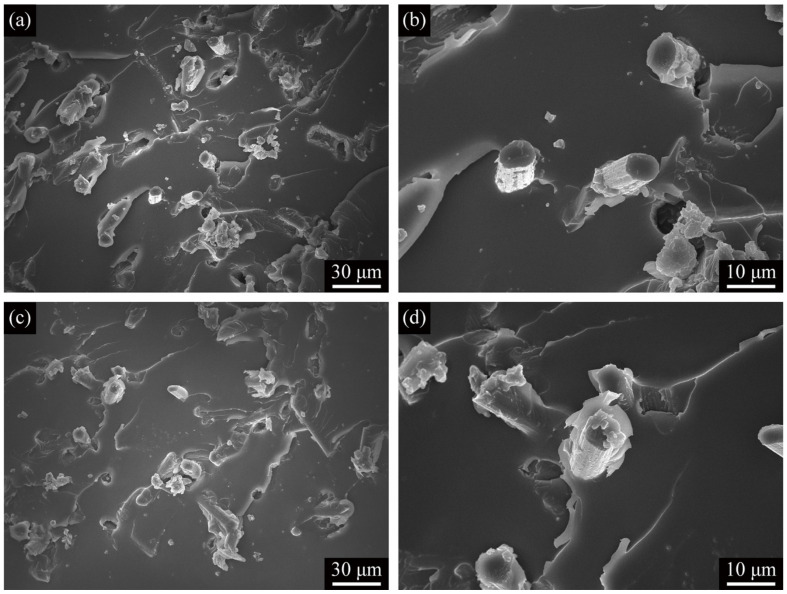
(**a**) Low-magnification and (**b**) high-magnification SEM images of SENB flexural fracture surfaces of C2/epoxy; (**c**) Low-magnification and (**d**) high-magnification SEM images of SENB flexural fracture surfaces of C2M/epoxy.

Figure 7 shows SENB flexural fracture surfaces of the C2/epoxy and C2M/epoxy composites. A large number of debonded reinforcements can be seen on both fracture surfaces. Still, unlike C2/epoxy (Figure 7a,b), the residual resin can be seen around the fibers in C2M/epoxy (Figure 7c,d), which indicates that the MXene functionalized ASCFs are more firmly bonded to the resin matrix.

As shown in Table 2, for neat epoxy, the flexural modulus of elasticity is equal to the tensile modulus of elasticity, but the flexural strength is much higher than tensile strength. Different from the three-point stress state of the bending test, any defects in the parallel section of the tensile specimen can lead to the failure of the specimen, so that the fracture surface may appear in any part of the parallel section of the specimen. However, the failure of the bending sample often occurs in the center of the sample under the action of the upper indenter, which is less affected by the defects than in the tensile test, that is why the flexural strength is greater than the tensile strength. Under the action of the reinforcing phase, although the flexural modulus and elastic modulus of the composite materials are slightly different, the trend and regular pattern of the two are consistent. In general, similar results can be seen in related reports on epoxy resin-based composites [39,62,63,64]. Similarly, by comparing the results of tensile strength, flexural strength and fracture toughness, it can be found that the same reinforcing phase content improves the two properties inconsistently, which is also related to different test methods. The results of the tensile and flexural test depend on the properties of the relatively large sample. Regardless of the nature of the sample, a single defect in the sample (such as agglomerate) can cause failure. In contrast, the sample damaged after the SEBN fracture test was carried out along the pre-crack, so it is not easily affected by a single defect. Therefore, the morphology of the tensile fracture surfaces of C2M/epoxy (Figure 6c,d) is completely different from that of the SENB flexural fracture surfaces (Figure 7c,d).

## 4. Conclusions

ASCFs with an average length of ~20 μm were prepared by grinding, sieving, washing and centrifuging nitric acid-treated CFs. A cross-scale enhancement of MXene functionalized ASCFs were prepared by immersing ASCFs in a solution with the Ti_3_C_2_T_x_ concentration of 1 mg mL^−1^. ASCF/epoxy composites with low-loads in 1 wt.%, 2 wt.%, 3 wt.% and 4 wt.% were prepared, and the mechanical properties of the composites are the best at a load of 2 wt.%. For comparison, the cross-scale reinforcements with ASCFs’ content of 2 wt.% were also composite with epoxy resin. The experimental results show that ASCFs functionalized by MXene form a stronger interface interaction with the epoxy matrix, which effectively improves the mechanical properties of the epoxy composite. In general, continuous CFs surface modification technology has been successfully applied to SCFs reinforced epoxy, providing a technical route with high-performance application prospects.

## Figures and Tables

**Table 1:** Dynamic mechanical properties of neat epoxy, ACSF/epoxy composites and MXene functionalized ACSF/epoxy composites.

Samples	Storage Modulus (MPa) (RT)	T_g_ (°C)
Neat epoxy	2209	131.3
C1/epoxy	2795	127.6
C2/epoxy	2994	126.3
C3/epoxy	3153	125.5
C4/epoxy	3273	124.0
C2M/epoxy	3044	126.9

**Table 2:** Mechanical and thermal properties of neat epoxy and its composites.

Samples	Tensile Strength (MPa)	Elastic Modulus (GPa)	Flexural Strength (MPa)	Flexural Modulus (GPa)	*K*_IC_ (MPa·m^1/2^)
Neat epoxy	70.5 ± 6.5	2.6 ± 0.3	119.1 ± 7.5	2.6 ± 0.2	0.74 ± 0.03
C1/epoxy	114.1 ± 1.4	3.7 ± 0.3	153.2 ± 8.7	3.2 ± 0.2	1.78 ± 0.03
C2/epoxy	124.6 ± 2.3	3.8 ± 0.2	165.7 ± 9.1	3.5 ± 0.3	2.14 ± 0.02
C3/epoxy	116.8 ± 1.1	3.8 ± 0.3	147.1 ± 8.9	3.3 ± 0.2	2.25 ± 0.04
C4/epoxy	104.3 ± 0.8	3.7 ± 0.1	129.8 ± 8.5	3.2 ± 0.4	2.32 ± 0.02
C2M/epoxy	141.2 ± 2.3	3.8 ± 0.4	199.3 ± 8.9	3.6 ± 0.3	2.34 ± 0.04

**Table 3:** Comparison of the tensile strength (σ), flexural strength (σ_fM_) and fracture toughness (*K_IC_*) of various composite systems, and the relative increments after incorporation of commercially available SCFs and MXene (Ti_3_C_2_T_x_).

Reinforcement Filler Content	σ, Gain in σ (%), (Matrix σ (MPa))	σ_fM_, Gain in σ_fM_ (%), (Matrix σ_fM_ (MPa))	*K*_IC_, Gain in *K*_IC_ (%), (Matrix *K*_IC_ (MPa·m^1/2^))	Reinforcement Filler and References
2 wt.%	124.6, 77, (70.5)	165.7, 39, (119.1)	2.14, 189, (0.74)	ASCFs, this work
2 wt.%	141.2, 100, (70.5)	199.3, 67, (119.1)	2.34, 216, (0.74)	MXene functionalized ACSFs, this work
17.5 Vf%	102.5, 53, (67.2)	-	-	SCFs [44]
2 wt.%	-	139.4, 28, (108.7)	~0.85, 35, (0.63)	SCFs [43]
3 wt.%	-	~130, 20, (108.7)	0.86, 36, (0.63)	
3 wt.%	-	135 ± 3, -, (-)	-	SCFs [59]
0.7 wt.%	~47, −11, (52.86)	89.69, 30, (69)	-	SCFs [60]
1 wt.%	55.61, 5.2, (52.86)	~72, 4, (69)	-	
0.2 wt.%	106.4, 51 (70.5)	157, 32, (119.1)	1.07, 45, (0.74)	Ti_3_C_2_T_x_ [39]
1 wt.%	76.1, 8, (70.5)	128.6, 8, (119.1)	1.41, 91, (0.74)	
1.2 wt.%	~66, 24.9, (~53)	-	-	Ti_3_C_2_T_x_ [61]

## Data Availability

The data presented in this study are available on request from the corresponding author.
